# Plant–microorganism–soil interaction under long-term low-dose ionizing radiation

**DOI:** 10.3389/fmicb.2023.1331477

**Published:** 2024-01-11

**Authors:** Guoqiang Zeng, Yingzi Wen, Chuyang Luo, Yihong Zhang, Fei Li, Chao Xiong

**Affiliations:** ^1^College of Nuclear Technology and Automation Engineering, Chengdu University of Technology, Chengdu, China; ^2^Applied Nuclear Techniques in Geosciences Key Laboratory of Sichuan, Chengdu University of Technology, Chengdu, China; ^3^Data Recovery Key Laboratory of Sichuan Province, Neijiang Normal University, Neijiang, China

**Keywords:** long-term low-dose ionizing radiation, adaptation to nuclear radiation, underground carbon and nitrogen cycle, plant–microorganism–soil interaction, symbiotic microbial effect

## Abstract

As the environmental nuclear radiation pollution caused by nuclear-contaminated water discharge and other factors intensifies, more plant–microorganism–soil systems will be under long-term low-dose ionizing radiation (LLR). However, the regulatory mechanisms of the plant–microorganism–soil system under LLR are still unclear. In this study, we study a system that has been stably exposed to low-dose ionizing radiation for 10 years and investigate the response of the plant–microorganism–soil system to LLR based on the decay of the absorbed dose rate with distance. The results show that LLR affects the carbon and nitrogen migration process between plant–microorganism–soil through the “symbiotic microbial effect.” The increase in the intensity of ionizing radiation led to a significant increase in the relative abundance of symbiotic fungi, such as Ectomycorrhizal fungi and Rhizobiales, which is accompanied by a significant increase in soil lignin peroxidase (LiP) activity, the C/N ratio, and C%. Meanwhile, enhanced radiation intensity causes adaptive changes in the plant functional traits. This study demonstrates that the “symbiotic microbial effect” of plant–microorganism–soil systems is an important process in terrestrial ecosystems in response to LLR.

## 1 Introduction

With the development of the nuclear industry and nuclear science, more and more ecosystems are suffering from ionizing radiation pollution. Especially after the Fukushima Daiichi Nuclear Disaster, Japan discharged a large amount of nuclear-contaminated water into the sea. The migration of these long half-life nuclides in the geochemical cycle will cause long-term radiation anomalies throughout the Earth’s ecosystems. Many regions (especially in Asia-Pacific and North America) will be under long-term low-intensity radiation (Chen et al., 2021). The increase in ionizing radiation in ecosystems carries out various effects, such as population extinction, biodiversity reduction, and changes in ecosystem structure and function (Geras’kin et al., 2008; Carolina et al., 2019; Mousseau and Møller, 2020; Elena, 2022). Especially in terrestrial ecosystems, there is a systematic correlation among plants, microorganisms, and soil. Aboveground vegetation, plant roots, soil physicochemical properties, and soil microorganisms are interconnected through nutrient cycle, energy flow, etc., which affect the structure and function of the whole terrestrial ecosystem (Castle et al., 2016; Song and Liu, 2018; Siddhartha and Karolina, 2022; Giovannetti et al., 2023; Song, 2023). Therefore, plant–microorganism–soil interaction is the main driving factor of carbon and nitrogen cycling (Delgado-Baquerizo et al., 2016). Some studies have shown that a certain dose of ionizing radiation will cause changes in plant functional traits and inhibit their growth and development, changes in the content, structure, and properties of organic and inorganic substances in soil, reduction of microbial diversity, and changes in ecosystem structure and function (Mohammad and Majed, 2019; Sergey et al., 2019; Cheptsov et al., 2021; Videvall et al., 2023). However, most of the studies on the ecological effects of ionizing radiation have mostly been carried out under laboratory conditions and usually based on a single species, excluding biological environmental factors and controlling non-biological factors, which makes these results have great limitations (Geras'kin, 2016). At present, little is known about the regulatory mechanisms of the plant–microorganism–soil system under long-term low-dose ionizing radiation (LLR). Therefore, it is of great significance to study the relationship of plant–microorganism–soil interaction in terrestrial ecosystems under long-term ionizing radiation.

In general, plants usually enhance their resistance by stimulating root-driven microbial communities when facing environmental stress and the same is true when subjected to nuclear radiation stress (Guesmi et al., 2022). Recent studies also indicated that under LLR conditions, plants tend to reduce aboveground biomass and invest more organic matter underground, thereby promoting the nutrient acquisition of the root-microbial system and improving the ecosystem’s adaptability to long-term nuclear radiation (Li et al., 2022; Cheng et al., 2023). According to previous studies, there are mainly two ways for plants to promote the nutrient acquisition of the root-microbial system, namely the “nitrogen mineralization effect” and the “symbiotic microbial effect,” which will lead to changes in soil carbon and nitrogen cycling and enzyme activity. The “nitrogen mineralization effect” refers to the phenomenon that plants increase the input of organic matter to the underground through the roots, which increases the number and activity of soil microorganisms, thus releasing more extracellular enzymes (such as proteases, cellulases, and lignin peroxidase), and then converting soil organic matter into inorganic forms of nutrients (such as ammoniacal nitrogen and nitrate nitrogen) that can be absorbed and utilized by plant roots, so as to improve plant nitrogen nutrition level and growth efficiency (Zang et al., 2016). The “symbiotic microbial effect” means to the phenomenon that plants increase the input of photosynthetic products and increase root exudates, which attract more symbiotic microorganisms (mainly mycorrhizal fungi), thus forming more hyphal networks extending into the soil, increasing the effective absorption area of plant roots, helping plants absorb more water and mineral nutrients, and enhancing plant adaptability (Harman et al., 2021). Therefore, two hypotheses are made as follows:

- The response of plant–microorganism–soil system to LLR is mainly based on “nitrogen mineralization effect.” In this case, the plant will release more root exudates, promote soil microorganisms to secrete extracellular enzymes to decompose soil organic matter, and obtain more inorganic nitrogen.
- The response of the plant–microorganism–soil system to LLR is mainly based on the “symbiotic microbial effect.” In this case, plants will rely more on microorganisms that are symbiotic with roots and obtain more mineral elements by increasing the number and activity of symbiotic microorganisms.

In order to discuss which interaction process is more realistic, in this study, we take a grassland ecosystem irradiated by radioactive nuclide thorium-232 for 10 years as the research object. By measuring the leaf functional traits and isotopic indicators of plants, as well as soil physicochemical properties, enzyme activities, and soil microbial community composition under different radiation doses, to verify the response of plant–microorganism–soil interaction under LLR. This study provides an important reference for coping with the impact of LLR on terrestrial ecosystems in future.

## 2 Materials and methods

### 2.1 Sample site introduction and sampling

The sample site is located in Chengdu, China, at the geographic coordinates of 108°8′11″E, 30°40′29″N. The soil type of this area is dark brown soil. The region has a subtropical monsoon climate, with an annual average precipitation of 1,150 mm, an average temperature of 17.1°C, and an average of 1,100 h of sunshine. A square-shaped thorium ore (an isotope of the radionuclide thorium-232) with a length, width, and height of 75 cm each is in the center of the sample site, exposing the surrounding ecosystem to radiation for more than a decade. Minerals are technically shaped and exposed to the surface of the soil. The radioactivity of the thorium mineral is about 2.93 × 10^6^ Bq. *Tradescantia fluminensis L.* is the overwhelmingly dominant group species in the region and has undergone multiple generations of reproduction in an LLR environment. Soil microorganisms also form stable communities adapted to the radiation environment (Carolina et al., 2019).

**Figure 1:**
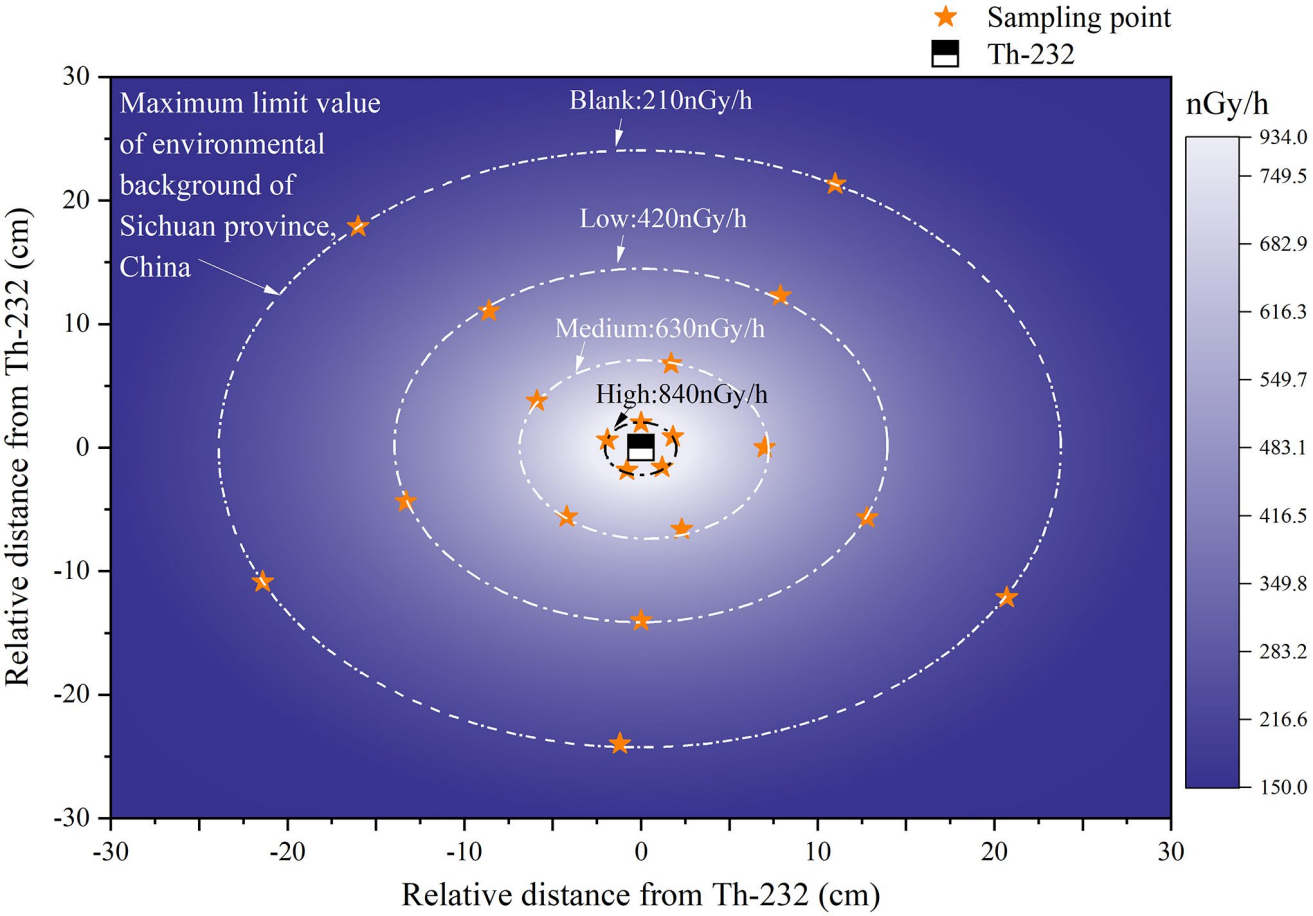
A coordinate system is established with the radiation source thorium-232 as the origin, and the schematic diagram of the surrounding absorbed dose rate and sampling points is shown. From inside to outside, pure white represents the maximum absorbed dose rate, and pure purple represents the minimum absorbed dose rate.

An HPGe portable gamma-ray spectrometer (trans-SPEC-DX-100 T) was used to measure the absorbed dose rate from thorium minerals at different distances. The highest absorbed dose rate at the periphery for thorium-232 was 910.964 ± 41.09 nGy/h and the lowest was 192.906 ± 5.05 nGy/h. Based on the attenuation of absorbed dose with distance, the sampling locations were set to three different gradients (2 ± 0.5 cm, 7 ± 0.5 cm, and 14 ± 0.5 cm). Their corresponding values were approximately four, three, and two times the maximum background-environmental radiation limit in Sichuan Province, which are known as high, medium, and low groups, respectively. In addition, samples were taken at the maximum limit of the environmental radioactivity background of the Sichuan Province as the blank group (Figure 1).

The collection time for plant and soil samples is 10 May 2021. Sixty healthy and growing plants were randomly selected and uprooted from four gradients. Samples of 0–20 cm topsoil were taken in five replicates at each radiation gradient. After removing stones, plant roots, and other sundry materials, the soil was mixed, bagged, sealed, and labeled and stored in a 0°C ice box at low temperature, transported to the laboratory, and stored at −20°C prior to analyses.

### 2.2 Plant functional traits

Leaf thickness, root length, and plant full length were measured with Vernier calipers. Leaf area was calculated by Photoshop pixel analysis method, which digitizes the leaf and automatically analyzes the selected leaf area of various shapes by using the Photoshop pixel analysis function, thus obtaining the leaf area size. The samples were air-dried to remove moisture in a fume hood, which then measured the mass by using the analytical balance.

Plants are ground to a 200-mesh powder with a mortar and pestle. The δ^13^C, δ^15^N, carbon, and nitrogen contents (g·g^−1^; %) of the samples were measured with a DELTA V Advantage Isotope Ratio Mass Spectrometer. Stable carbon isotope discrimination was calculated with the following Equation (1):


(1) ΔC13=δa−δp1+δp

Where δ_a_ is the δ^13^C value of CO_2_ in the atmosphere and δ_p_ is the δ^13^C value of plant samples. The phosphorus content (g·g^−1^; %) of the samples was measured with an AxiosmAX Wavelength Dispersive X-ray Fluorescence (WDXRF) spectrometer from PANalytical B.V. (Shen, 2012). The spectrometer uses a Rh-target X-ray tube, set at 60 kV, 125 mA current, and 20 min measurement time. Quantitative analysis software is Super Q 4.0. The values in the graph are the mean ± standard error.

### 2.3 Physical and chemical properties of soil

The samples were extracted with a potassium chloride solution, and then, the soil ammonium nitrogen content was determined by indophenol blue colorimetry, and the determination of soil nitrate nitrogen content by dual-wavelength colorimetry. The sum of NH+ 4-N and NO- 3-N extracted from the cultured sample and the resin bag below the soil column was subtracted from the sum of NH+ 4-N and NO- 3-N extracted from the initial sample and divided by the incubation days to obtain the nitrogen mineralization rate of the sample (Miransari and Mackenzie, 2010).

Lignin peroxidase (LiP) oxidizes veratrylalcohol to form veratrylaldehyde. The absorbance at 310 nm was measured to calculate LiP activity (Guan, 1982). β-Glucosidase (β-GC) catalyzes the formation of p-nitrophenol from p-nitrobenzene-β-D-glucopyranoside. The absorbance of the p-nitrophenol at 405 nm was measured to calculate β-GC activity (Dick et al., 2013). In an alkaline environment, alkaline phosphatase (AKP) was used to catalyze the hydrolysis of disodium phenyl phosphate to produce phenol and disodium hydrogen phosphate, and AKP activity was calculated by measuring the amount of phenol produced (Guan, 1982). The NH_3_-N produced by urease (UE) hydrolysis of urea was determined by indophenol blue colorimetry to calculate UE activity (Guo et al., 2012). The content of reducing sugar produced by cellulase (CL) catalytic cellulose degradation was determined by anthrone colorimetry to calculate CL activity (Guan, 1982). Under alkaline conditions, alkaline protease (ALPT) can hydrolyze casein to produce tyrosine, which reduces phosphomolybdic acid compounds to produce tungsten blue. The absorbance of the latter at 680 nm was measured to calculate ALPT activity (Geisseler and Horwath, 2008).

### 2.4 Soil microbial DNA extraction, PCR amplification, and sequencing

Soil microbiome DNA extraction was performed with the PowerSoil DNA Isolation Kit (MoBio Laboratories, Carlsbad, CA, United States) following the specification. For extraction, 0.25 g (fresh weight) of each soil sample was weighed. The quality and concentration of the DNA extracted were measured with 1% agarose gel electrophoresis and spectrophotometry. The mean DNA concentration was 212.5 ng/μl. The samples were stored at −20°C for later experiments.

PCR amplification and sequencing were used to identify the bacterial and fungal species. The primers 338F (5′-ACTCCTACGGGAGGCAGCAG-3′) and 806R (5′-GGACTACNNGGGTATCTAAT-3′) were used to amplify the v.3-v.4 region of the 16S rRNA gene of bacteria. The primers ITS1 (5′-CTTGGTCATTTAGAGGAAGTAA-3′) and ITS2 (5′-TGCGTTCTTCATCGATGC-3′) were used to amplify the ITS1 region of the ITS gene of fungi. Barcode sequences 8 bp in length were added to the 5′ ends of the upstream and downstream primers to differentiate the different samples. The PCR system for both 16S rRNA and ITS (total system of 25 μL)were as follows: 12.5 μL 2xTaq Plus Master Mix, 3 μL BSA (2 ng/μL), 1 μL forward primer (5 μM), 1 μL reverse primer (5 μM), 2 μL DNA (30 ng), and 5.5 μL ddH_2_O to equal a final volume of 25 μL. The thermocycling conditions of the 16S rRNA reaction were as follows: pre-denaturation at 94°C for 5 min; 30 cycles of denaturation at 94°C for 30 s, annealing at 50°C for 30 s, and extension at 72°C for 60 s; and final extension at 72°C for 7 min. The thermocycling conditions of the ITS reaction were as follows: pre-denaturation at 94°C for 5 min; 34 cycles of denaturation at 94°C for 30 s, annealing at 55°C for 30 s, and extension at 72°C for 60 s; and final extension at 72°C for 7 min. PCR products were detected by 1% agarose gel electrophoresis to detect the size of amplified target bands and purified by the Agencourt AMPure XP Nucleic Acid Purification Kit.

The PCR products were used to construct microbial diversity sequencing libraries, and paired-end sequencing was performed using the Illumina Miseq PE300 high-throughput sequencing platform. The raw sequencing sequences were uploaded to the NCBI SRA database. The origin sequences were split by the Barcode sequence according to the QIIME2 software (Bolyen et al., 2019), and the data were filtered and spliced by the Pear (v2.0.9) software. Remove low scores of 20, sequences with ambiguous bases, and primer mismatch sequences. The minimum overlap was set to 10 bp, and the mismatch rate was 0.1 when splicing. After splicing, the Vsearch (v2.8.1) software was used to remove sequences with a length of less than 230 bp, and the chimeric sequences were removed by comparing with the Gold Database using the uchime method (Rognes et al., 2016). The VSEARCH (v2.8.1) software program was used to cluster high-quality sequences with operational taxonomic units (OTUs), with the sequence similarity threshold at 97% (Edgar, 2013). Silva and Unite databases were used to obtain the species classification data corresponding to each OTU. The Ectomycorrhizal fungi were divided using FungalTraits (Põlme et al., 2020).

## 3 Results

### 3.1 Changes in soil microbial community

**Figure 2:**
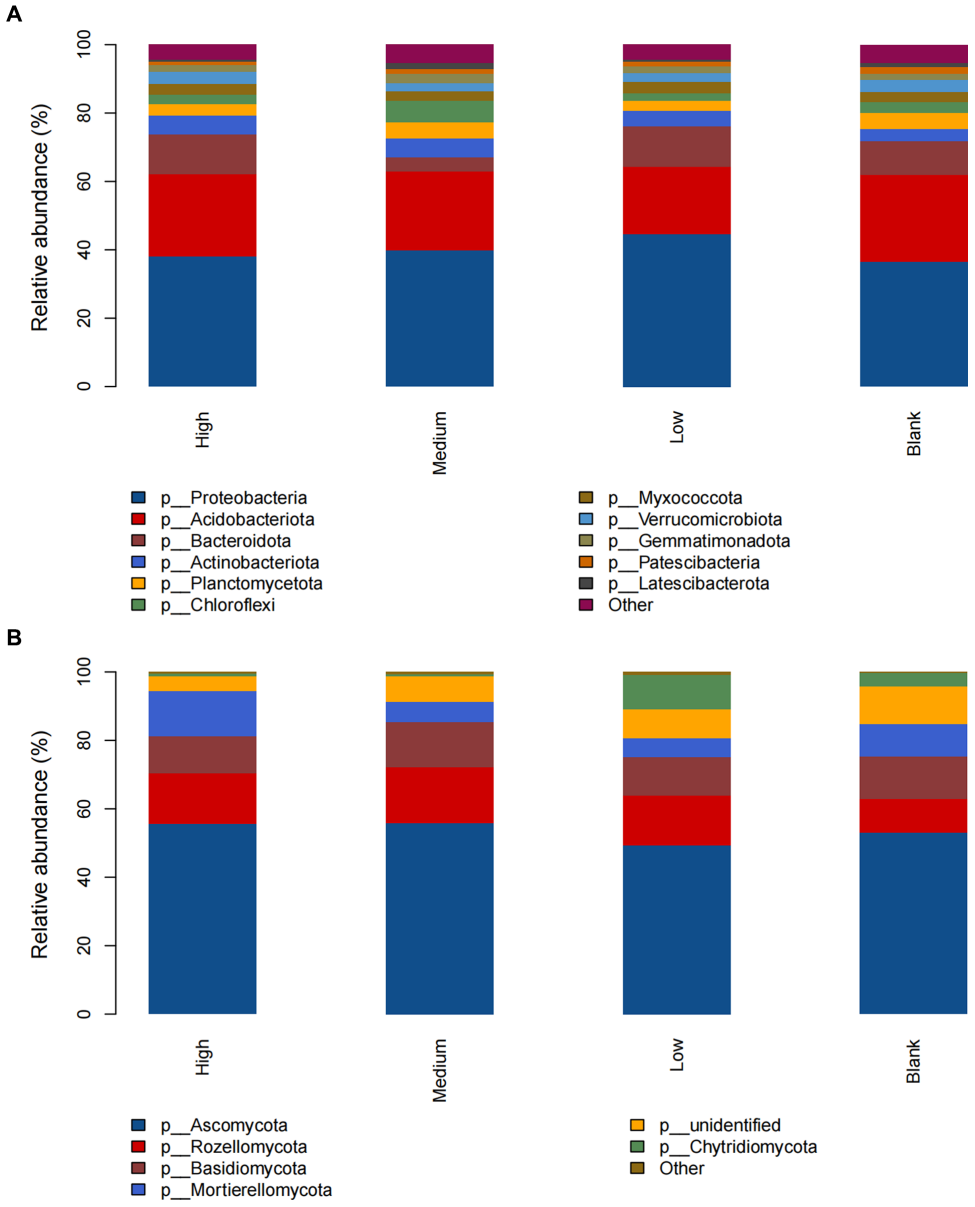
The composition of soil **(A)** bacteria and **(B)** fungi at the phylum level under different radiation intensities.

At the phylum level of the bacterial community, the dominant groups in the high, medium, low, and blank groups were *Proteobacteria* and *Acidobacteriota*; the relative abundance of *Bacteroidota* in the medium group was significantly lower than that of the other groups, but the relative abundance of *Chloroflexi* was significantly higher than that of the other groups; the relative abundance of other phyla did not change significantly (Figure 2A). At the phylum level of the fungal community, the dominant groups of the high, medium, low, and blank groups were *Ascomycota*, and the relative abundance of *Rozellomycota* in the high, medium, and low groups was higher than that of the blank group; with the increase in radiation intensity, the relative abundance of *Mortierellomycota* in the medium and low groups was lower than that of the blank group, while that in the high group was significantly higher than that of the blank group; the relative abundance of *Chytridiomycota* in the low group was higher than that of the blank group, while that in the high and medium groups was significantly lower than that of the blank group; and the relative abundance of other phyla did not change significantly (Figure 2B).

### 3.2 Changes in plant functional traits

**Figure 3:**
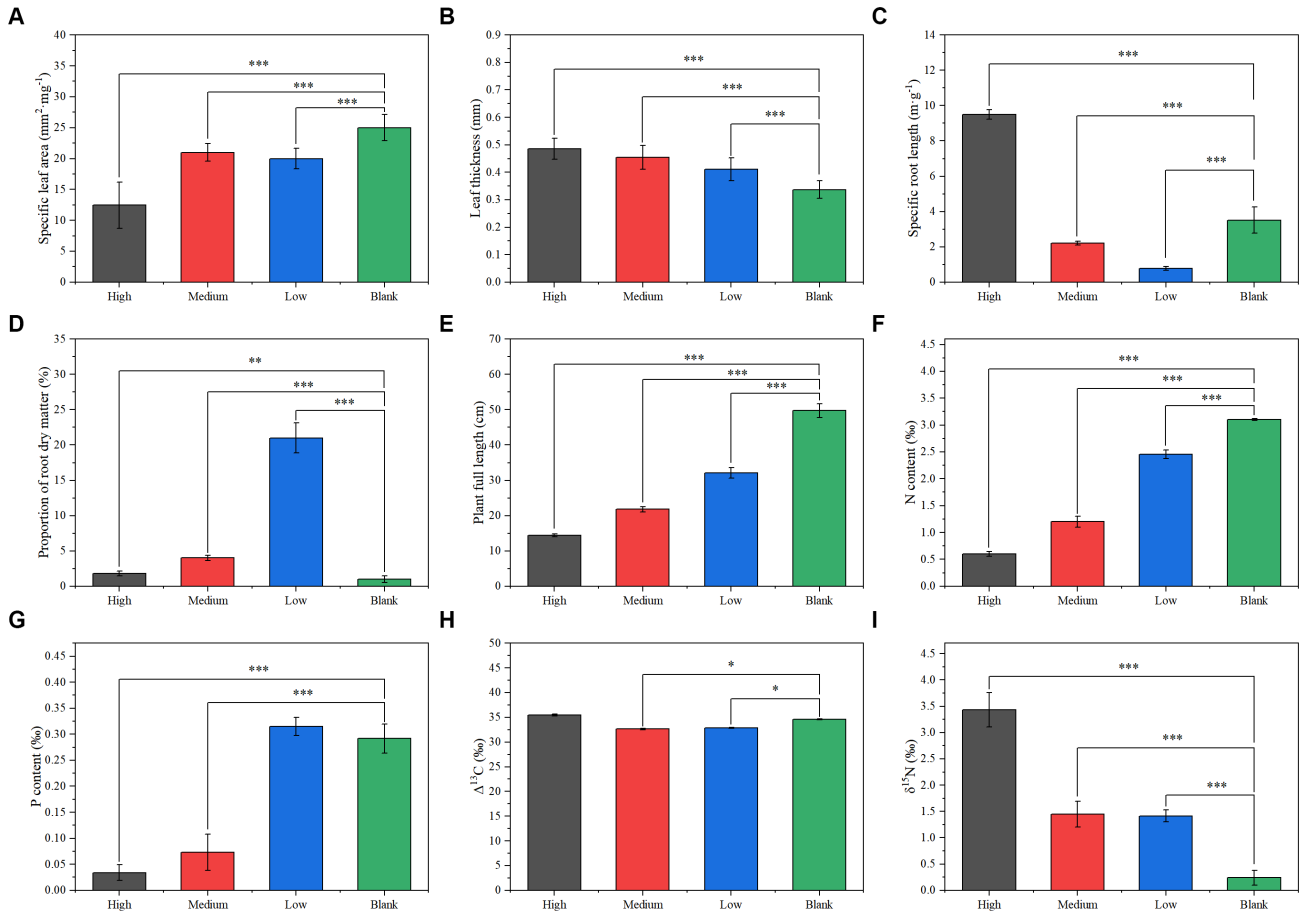
Plant functional traits under different radiation gradients: **(A)** specific leaf area, **(B)** leaf thickness, **(C)** specific root length, **(D)** root dry matter ratio, **(E)** plant height, **(F)** nitrogen concentration, **(G)** phosphorus concentration, **(H)** Δ^13^C, **(I)** δ^15^N. Significance: ^*^<0.05; ^**^<0.01; ^***^<0.001.

There were no significant differences in plant composition, diversity, and biomass in the sample plots at different radiation intensities. However, long-term ionizing radiation had a significant effect on plant functional traits. Compared with the blank group, the specific leaf area of the long-term radiation group decreased significantly, and the lowest was in the high group (Figure 3A). While the leaf thickness increases gradually with the increase in radiation intensity (Figure 3B). The specific root length of the low and medium groups was significantly lower than that of the blank group, but the specific root length of the high group was significantly higher than that of the blank group (Figure 3C). The proportion of root dry matter of the low group was significantly higher than that of the blank group, while the proportion of root dry matter of the medium group was significantly lower than that of the low group, and that of the high group was significantly lower than that of the medium group (Figure 3D). The plant full length decreases gradually with the increase in radiation intensity (Figure 3E). The plant nitrogen content decreases gradually with an increase in radiation intensity (Figure 3F). The plant phosphorus content of the low group did not differ significantly from that of the blank group, but the plant phosphorus content of the medium and high groups decreased significantly in turn (Figure 3G). The Δ^13^C values of the low and medium groups were significantly lower than that of the blank group, but the Δ^13^C value of the high group did not differ significantly from that of the blank group (Figure 3H). The Δ^15^N values of the low and medium groups were significantly higher than that of the blank group, and the Δ^15^N value of the high group was significantly higher than that of the low and medium groups (Figure 3I).

### 3.3 Effects of ionizing radiation

**Figure 4:**
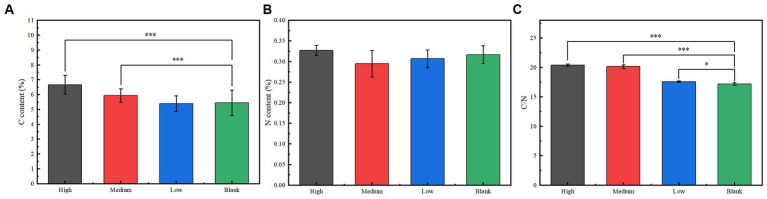
Soil **(A)** C content, **(B)** N content, and **(C)** C/N ratio under different radiation gradients. Significance: ^*^<0.05; ^**^<0.01; ^***^<0.001.

**Figure 5:**
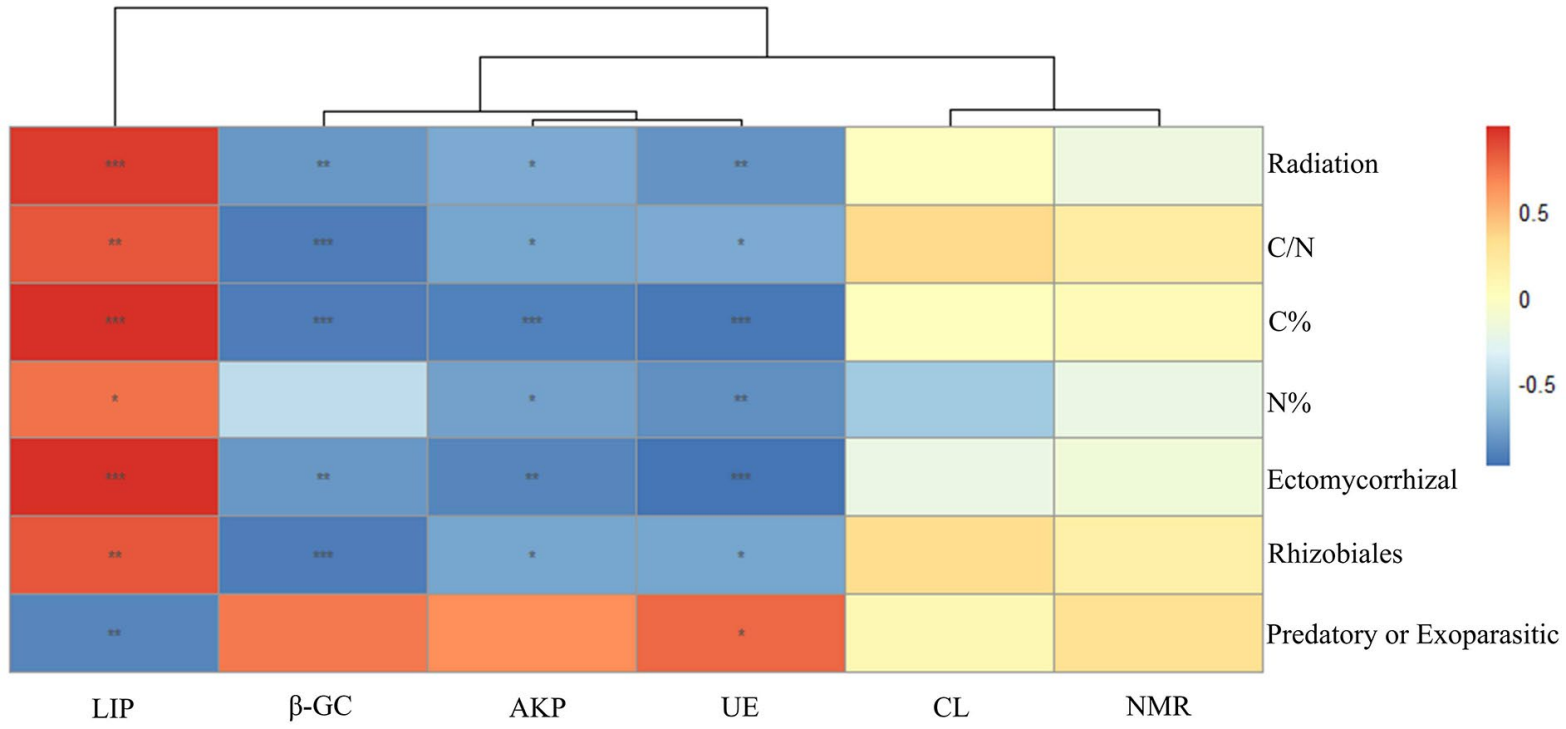
Relationships of soil nitrogen mineralization rate (NMR), soil lignin peroxidase (LiP), cellulase (CL), β-glucosidase (β-GC), alkaline phosphatase (AKP), and urease (UE) with radiation intensity, C content, N content, C/N ratio, and the abundance of Ectomycorrhizal fungi, Rhizobiales, and *Predatory* or *Exoparasitic*. Color depth represents the level of correlation. Significance: ^*^<0.05; ^**^<0.01; ^***^<0.001. Distance algorithm: Bray–Curtis, clustering method: Pearson.

No significant differences were found in soil moisture, pH (around 7.5), light, and other environmental factors among plots under different radiation intensities. This indicated that the adaptive changes in plant functional traits and microbial communities are caused by ionizing radiation itself, rather than other environmental changes caused by ionizing radiation. The C% in the soil of the low group was not significantly different from that of the blank group, while that of the medium group was significantly higher than that of the blank group, and that of the high group was significantly higher than that of the medium group; there was no significant difference in soil N% among groups; the C/N ratio of the low group was significantly higher than that of the blank group, and that of the high and medium groups were significantly higher than that of the low group (Figure 4). Soil nitrogen mineralization rate had no significant correlation with radiation intensity, C/N ratio, C%, N%, and abundance of Ectomycorrhizal fungi, Rhizobiales, and *Predatory* or *Exoparasitic* (Figure 5). β-GC, AKP, and UE activities had significant negative correlations with radiation intensity, C/N ratio, C%, and abundances of Ectomycorrhizal fungi and Rhizobiales (Figure 5). AKP and UE activities had significant negative correlations with N% (Figure 5). LiP activity had significant positive correlations with radiation intensity, C/N ratio, C%, and N%, and abundances of Ectomycorrhizal fungi and Rhizobiales, but had significant negative correlations with an abundance of *Predatory* or *Exoparasitic* (Figure 5). CL activity had no significant correlation with radiation intensity, C/N ratio, C%, N%, and abundance of Ectomycorrhizal fungi, Rhizobiales, and *Predatory* or *Exoparasitic* (Figure 5).

## 4 Discussion

### 4.1 Nitrogen mineralization effect versus symbiotic microbial effect

When the nitrogen mineralization effect occurs, then must accompanied by phenomena such as an increase in the rate of nitrogen mineralization, an increase in the activities of enzymes related to nitrogen mineralization, a decrease in soil organic carbon due to the accelerated decomposition of organic matter, and a decrease in total soil nitrogen but an increase in the amount of the available nitrogen (Kuzyakov and Xu, 2013). At the same time, the specific leaf area and plant biomass should increase (Abalos et al., 2018), and the δ^15^N of the plant should not change significantly (Song et al., 2020). However, our study found that there is no significant change in soil N mineralization rate in relation to radiation intensity, C/N ratio, C%, and N%, and the activities of CL, β-GC, AKP, and UE are either significant negative correlate or non-significant correlate with radiation intensity, C/N ratio, C%, and N%. In addition, with the increase in radiation intensity, soil N% did not change significantly, while C% not only did not decrease but increased significantly. The specific leaf area and plant biomass not only did not increase, but instead decreased, while the δ^15^N of the plant increased significantly. Therefore, the hypothesis of the “nitrogen mineralization effect” is not valid.

When the symbiotic microbial effect occurs, the symbiotic microorganism, mainly Ectomycorrhizal fungi, in order to obtain nutrients such as nitrogen and phosphorus from difficult-to-break-down organic matter such as lignin in exchange to plants, usually release LiP to transform difficult-to-break-down plant residues, simultaneously promoting soil carbon and nitrogen sequestration (Zak et al., 2019; Argiroff et al., 2022). Moreover, because the δ^15^N of the nitrogen element given to the plant by the mycorrhizal fungi is high, causing the δ^15^N of the plant is also high (Song and Zhou, 2021). We found that LiP activity has significant positive correlations with radiation intensity, C/N ratio, C%, N%, and abundance of Ectomycorrhizal fungi and Rhizobiales, and plant δ^15^N increase with increasing radiation intensity, which is consistent with the hypothesis of the “symbiotic microbial effect.” In addition, the symbiotic microbial effect can also explain some physiological changes in plants to adapt to long-term ionizing radiation. For example, the plants in the low group improve their damaged nutritional status due to radiation by enhancing the nutrient absorption ability of the root system, which makes the specific root length of the plants in the low group significantly lower than that of the blank group, while the root dry matter proportion is significantly higher than that of the blank group (Li et al., 2022). Moreover, the abundance of Ectomycorrhizal fungi in the high group is 6 times that of the medium group, 13 times that of the low group, and 0 in the blank group. This indicates that under long-term ionizing radiation stress, plants transfer the main task of absorbing nutrients to symbiotic microorganisms mainly composed of Ectomycorrhizal fungi, resulting in significantly longer specific root length than that of the blank group, while the proportion of root dry matter is significantly lower than that of the blank group (Li et al., 2022; Cheng et al., 2023).

### 4.2 Response of plant–microbial–soil system to ionizing radiation

The radiation intensity in this study is not enough to change the dominant groups of soil microorganisms, but the relative abundance of some microbial phyla still changed under different irradiation intensities. The bacterial abundance composition is relatively stable, and only the abundance of *Bacteroidota* and *Chloroflexi* in the medium group changed greatly. In the fungi composition, compared to the blank and low groups, *Chytridiomycota* is significantly reduced in the medium and high groups. *Chytridiomycota* is a kind of transgenic parasitic fungi of various plants, and its produced zoospores are mediators for the transmission of some viruses in the soil (James et al., 2006; Kaczmarek and Boguś, 2021). A reduction in the relative abundance of *Chytridiomycota* reduces soil-borne diseases and thus indirectly promotes plant growth. The relative abundance of *Mortierellomycota* in the high group is significantly higher than that in the other groups. Some types of *Mortierella alpina* can significantly increase the content of soil-dissolved organic carbon, resulting in a significantly higher C% in the high group compared to the other groups (Wang et al., 2022).

In order to cope with the adverse effects of the radiation environment, plants under different radiation intensities have made adaptive changes to the varying radiation intensity, such as reducing specific leaf area, increasing leaf thickness, and shortening plant length (Li et al., 2022). In this case, the plants do not have excess root exudates to stimulate soil microorganisms for nitrogen mineralization. However, while making these stress-resistance responses, plants also reduce their nutrient absorption ability, making their nitrogen and phosphorus content lower. At this time, plants usually turn to rhizosphere microorganisms (especially root symbiotic microorganisms) to obtain nutrients such as nitrogen and phosphorus (Chai and Schachtman, 2022). Under the influence of this symbiotic microbial effect, the abundance of Ectomycorrhizal fungi increased significantly with the increase in radiation intensity. Because Ectomycorrhizal fungi form a biofilm on the root surface, this biofilm not only helps plants resist the invasion of pathogens, toxins, and drought (Peter et al., 2016; Vincent and Declerck, 2021; Zheng and Song, 2022) but also promotes soil carbon sequestration by sequestering as microbial residues in the soil for a long period of time because of the difficulty of decomposition of its constituents (Liang et al., 2017; Liang, 2020; Song et al., 2023). Moreover, Rhizobiales can also exchange nutrients with plants and have the ability to promote soil carbon sequestration (Song et al., 2020). Therefore, with the increase in radiation intensity, the abundance of Ectomycorrhizal fungi and Rhizobiales also increase, thereby increasing soil C% and increasing the C/N ratio (Wallander et al., 2011). Ionizing radiation also inhibited *Predatory* or *Exoparasitic*, thus indirectly stabilizing ecosystem productivity (Liu et al., 2022), which, in turn, affects soil nutrient changes (Ning et al., 2021). In addition, the Δ^13^C value reflects the water use efficiency of plants (Song and Liu, 2020), and the Δ^13^C values of the low and medium groups are significantly lower than that of the blank group, but the Δ^13^C value of the high group do not differ significantly from that of the blank group, indicating that the symbiotic microbial effect can restore the water use efficiency of plants damaged by radiation. In summary, we believe that root symbiotic microorganisms are important research objects to regulating the function of plant–microorganism–soil systems under LLR and coping with global ionizing radiation pollution in future.

## 5 Conclusion

Compared with the nitrogen mineralization effect, the symbiotic microbial effect is more consistent with the actual situation of plant–microorganism–soil interactions under LLR. The symbiotic microbial effect changes the carbon and nitrogen migration processes and thus helps plants cope with the nutrient status damaged by radiation. Under the symbiotic microbial effect, radiation promotes the growth of Ectomycorrhizal fungi and Rhizobiales and inhibits the growth of *Predatory* or *Exoparasitic*, which increases soil C% and C/N ratio. The greater the radiation intensity, the greater the effect. This study believes that long-term ionizing radiation has a significant interaction with plant–microorganism–soil system. Root symbiotic microorganisms (especially Ectomycorrhizal fungi) are important microbial groups to cope with LLR in future, and symbiotic microbial effect is an important process affecting the response of terrestrial ecosystems to LLR.

## Data availability statement

The datasets presented in this study can be found in online repositories. The names of the repository/repositories and accession number(s) can be found at: https://www.ncbi.nlm.nih.gov/, PRJNA950186.

## Author contributions

GZ: Writing – original draft. YW: Writing – original draft. CL: Validation, Writing – review & editing. YZ: Investigation, Writing – original draft. FL: Methodology, Visualization, Writing – original draft. CX: Conceptualization, Validation, Writing – review & editing.
